# Clonal diversity of *Haemophilus influenzae carriage* isolated from under the age of 6 years children

**DOI:** 10.1186/s13104-019-4603-7

**Published:** 2019-09-11

**Authors:** Fahimeh Shooraj, Bahman Mirzaei, Seyed Fazlollah Mousavi, Farzaneh Hosseini

**Affiliations:** 1 Department of Microbiology, Pasteur Institute of Iran, Tehran, Iran; 2 Department of Microbiology and Virology, School of Medicine, Zanjan University of Medical Sciences, Zanjan, Iran; 3 Department of Microbiology, Faculty of Biology, Islamic Azad University, North Branch, Tehran, Iran

**Keywords:** *Hemophilus influ*enzae, Antimicrobial resistance, PFGE, Genomic analysis

## Abstract

**Objectives:**

Pharyngeal carriers such as *H*. *influenzae* seem to constitute the only reservoir and probably the only transmission vehicle of the invasive disease. The aims of this study were to estimate the prevalence of *H. influenzae* carriage, to characterize antibiotic susceptibility, and to explore genetic diversity of *H. influenzae* isolates. Sampling was carried out as nasopharynx swabs among children less than 6 years old volunteers. After traditional biochemical tests, isolates were confirmed by targeting *omp6* sequence. Following the susceptibility tests, genomic diversity of strains was analyzed by Pulsed-Field Gel Electrophoresis procedure.

**Results:**

Out of 328 nasopharynx swabs, 73 strains were identified as H. *influenzae*. Among *H. influenzae* isolates, resistance to chloramphenicol (42%) and ampicillin (43%) was observed. Levofloxacin is the most effective antibiotic and the least effect belonged to tetracycline. By genomic analysis of selected *H*. *influenza*, 28 PFGE patterns were achieved among which 11 patterns included at least 2 strains. All strains clustered into 25 different clones. The dendrogram analysis of the isolated *H*. *influenzae* strains showed that some of these strains had a clonal relationship and common genetic origin. According to our results, antibiotic resistance didn’t show any significant correlation with the clonality of strains.

## Introduction

*Haemophilus influenzae* as an opportunistic pathogen is responsible for one-third of bacterial pneumonia cases among children of 4 months to 4 years old [1]. Most healthy *H. influenzae* pharyngeal carriers are children in countries who have not vaccinated against of *H. influenzae*. According to reports, the rate of carriage of *H. influenzae* in infants are about 20% and in children 5 to 6 years old are > 50% [2]. Antibiotic resistance causes therapeutic failures and continues existence of resistant strains and so further spread of the disease in medical centers [3–7]. Since 1987, *amoxicillin*-*clavulanate, cefuroxime axetil and cefpodoxime proxetil remain active and rates of resistance to trimethoprim*-*sulfamethoxazole, chloramphenicol, cefaclor, tetracycline, azithromycin and clarithromycin are generally low. H. influenza related infections was treated by single or combination of mentioned drugs* [8]. Growing resistance to antibiotics such as co-trimoxazole, ampicillin, tetracycline, amoxicillin, cefixime, and ciprofloxacin as a *H. influenzae* associated infections drug of choice is worrying [9–11]. After introducing of the pentavalent vaccine at the end of 2014 in Iran, to national immunization program 90%–95% preventable potential to *H. influenza* related infections was estimated [12]. The survival of resistant strains usually results in the fast spread of the resistant types of bacteria and finally the spread of diseases. Therefore, it is very useful to study the trend of clonal spread and trace the origin of strains in epidemiologic studies [13–15]. Among methods used in studying the clonal spread and genetic similarity between the strains of bacteria, we can refer to Pulsed Field Gel Electrophoresis (PFGE), which is used as a golden standard method for all bacteria such as *H. influenzae* [16]. This technique can show the vertical transmission of resistance genes among the bacteria [17–19]. Studying the pattern of genome can show the genetic similarity between strains susceptible and resistant to antibiotics [16]. No epidemiologic study has been carried out in Iran to determine the spread of *H. influenzae* carriers compared to antibiotics susceptibility of them. The aims of this study were to estimate the prevalence of *H. influenzae* carriage, to characterize antibiotic susceptibility, and to explore genetic diversity of *H. influenzae* isolates. Thus, this study presents exclusive epidemiologic information about *H. influenzae* strains isolated from children in Tehran, Iran. This can be helpful for choosing an appropriate therapy to remove infections caused by this bacterium.

## Main text

### Materials and methods

#### Nasopharyngeal samples

Sampling was done from 328 volunteers within 10 months from June to the late March, 2016. Samples were collected from children less than 6 years old from six medical centers for children in Tehran including Children’s Medical Center and Ameneh, Shobeir, Torkamani and Roghayyeh nurseries. Samples swabs were immediately put into stewart transport medium and transferred to lab during 2 to 3 h. Samples were then cultured in chocolate agar containing 260 µg/ml bacitracin.

#### Isolation of *H. influenzae* strains

Prior identification of suspected *H. influenzae* colonies was accomplished basing on the scheme utilized in Diagnostic Microbiology Textbook including ureas, indole, motility, oxidase and catalase [20].

#### Reconfirmation of suspected isolates by PCR

Suspected growth colonies were screened based on the targeting a conserved sequences (*ompP6*) were determined in both encapsulated and non-encapsulated *H. influenzae* [21]. Following the extraction of crude DNA taking advantage of a commercial high pure extraction kit (Roche Diagnostics GmbH, Mannheim, Germany) quantity of extracted DNA was adjusted to 500 ng μl-1. Amplification was done on Gene Amp PCR system (Applied Biosystem, USA) by uniplex PCR method in total volume 25 μl containing; 12 μl master amplicon (Biolab, New England, UK), 1 pmol of each forward and reverse primers, a 1 μl of crude DNA as the template and 10 μl distilled water. Using program an initial cycle of denaturation 94 °C for 3 min, followed by 25 cycles of 94 min, for 1 min, 60 °C for 1 min and 72 °C for 1 min with a terminal extension for 10 min. Resulting PCR were visualized with 1% agarose gel (KBC, Max Pure agarose, Spain). *H. influenzae* ATCC 9007 and *Pseudomonas aeruginosa* ATCC 27853 were served as positive and negative strains (Additional file 1).

#### Assessing antibiotic sensitivity

**Fig. 1:**
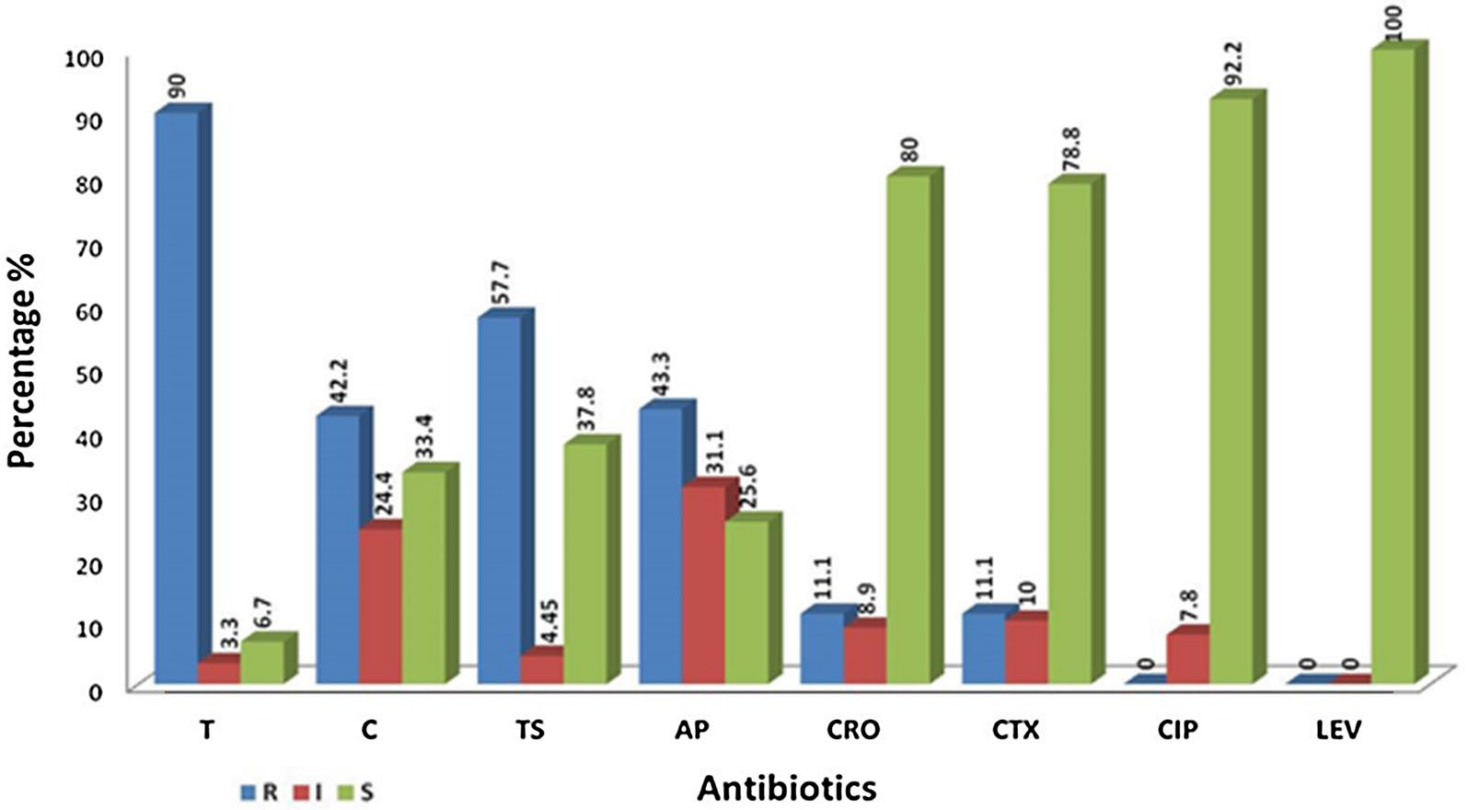
Susceptibility patterns of nasopharyngeal isolates basing on the minimum bactericidal concentration criteria. T, tetracycline; C, chloramphenicol; TS, co-trimoxazole; AP, ampicillin; CRO, ceftriaxone; CTX, cefotaxime; CIP, ciprofloxacin; LEV, levofloxacin

Susceptibility testing was done based on the CLSI recommended guidelines [3] using specific antibiotics disks (BD BBLTM Sensi DiscTM) include Levofloxacin 5 µg, Ciprofloxacin 5 µg, Ceftriaxone 30 µg, Cefotaxime 30 µg, Co-trimoxazole 23/75 µg, Chloramphenicol 30 µg, Ampicillin 10 µg, and Tetracycline 30 µg on the Hemophilus test medium (HTM) containing Muller hinton agar supplemented by 15 µg/ml NAD and 5 µg bovine hematin considering Clinical Laboratory Standards Institute (Fig. 1). Strains resistant to ampicillin and chloramphenicol and the strain showed intermediate resistance were selected to determine the Minimal Inhibitory Concentration (MIC) of ampicillin and chloramphenicol. Microdilution Broth was used to determine MIC [3]. *H. influenzae* ATCC 49247 was used as the standard control strain.

#### Studying molecular epidemiology

For studying molecular epidemiology of the collected strains, Pulsed Field Gel Electrophoresis (PFGE) was used. The protocol was described previously by Saito et al. [16]. Briefly, the plugs were digested with *Sma*I (30U) within 6 h under 25 °C. The program of CHEF-DR III apparatus (Bio-Rad, Richmond, Calif.) was adjusted for the movement of digested bands as the pulse time of 0.5 to 10 s for 16 h and 10 to 17 s for 4 h under the temperature of 4 °C, the voltage of 6 V/cm^2^ and the angle of 120°. The analysis of bands was performed by Gel Compare II software (Applied Maths, Belgium). *Salmonella branderup* H9812 was used as size marker.

### Results

#### Identifying and confirming samples containing *H. influenzae*

In this research, 328 collected swabs were evaluated based on biochemical tests in which 108/328 samples (33%) were positive for *H. influenzae.* consequently, considering *omp6* targeted sequence, 73/328 (22%) strains precisely reconfirmed. Mentioned strains were adjusted to susceptibility testing and clonal diversity assessment as well.

#### Antibiogram and MIC

According to CLSI instructions, the resistance pattern of confirmed strains (n = 73) to selected antibiotics was assessed and reported based on the percentage of resistant, intermediate and susceptible strains (Fig. 1). As observed in Fig. 1, the most effective antibiotics were levofloxacin 100%, ciprofloxacin 92%, ceftriaxone 80%, cefotaxime 79%, co-trimoxazole 38%, chloramphenicol 33%, ampicillin 26%, and tetracycline 7% respectively. Accordingly, the most effective drug against these strains was levofloxacin and the least effect belonged to tetracycline. MIC of resistant and intermediate analysed strains for two assessed antibiotics (ampicillin and chloramphenicol) was cheeked followed by Kirby–Bauer break point determination as well (Additional file 2). Among 57 strains for which the MIC of ampicillin was studied, 20 strains were identified by disk diffusion as intermediate and 37 were resistant. Among them 38 strains were susceptible by MIC. Among 49 strains which the MIC of chloramphenicol was studied for them, using disk diffusion method, 20 strains were identified as intermediate and 29 were resistant. According to MIC, 47 strains were susceptible by MIC. Considering the susceptibility pattern via disc diffusion procedure all isolates were determined as multidrug resistant (MDR) strains. Detailed data listed in Table 1.

**Table 1:** Frequency of Multi drug resistant H. *influenza* isolates considering the Minimum bactericidal break points

Source of collected samples	Number of Isolates resistant to ≥ 2 antibiotics (N = 73)	Multi drug resistant isolates				
		Two ABs	Three ABs	Four ABs	Five ABs	Six ABs
Childrens Medical Center	15 (20%)	8 (11%)	3 (4%)	3 (4%)	1 (1%)	–
Ameneh Nursery	15 (20%)	8 (11%)	3 (4%)	3 (4%)	–	1 (1%)
Shobeir Nursery	26 (36%)	5 (7%)	8 (11%)	9 (12%)	2 (3%)	2 (3%)
Torkamani Nursery	9 (12%)	5 (7%)	3 (4%)	–	–	1 (1%)
Roghayyeh Nursery	8 (11%)	4 (5%)	2 (3%)	2 (3%)	–	–
Total (%)	73 (100%)	30 (41%)	19 (26%)	17 (23%)	3 (4%)	4 (6%)

Total percentage were specified in the end of the each column

N, number of reconfirmed *H. influenza* strains; ABs, antibiotics

#### Genomic model of nasopharynx strains by PFGE

**Fig. 2:**
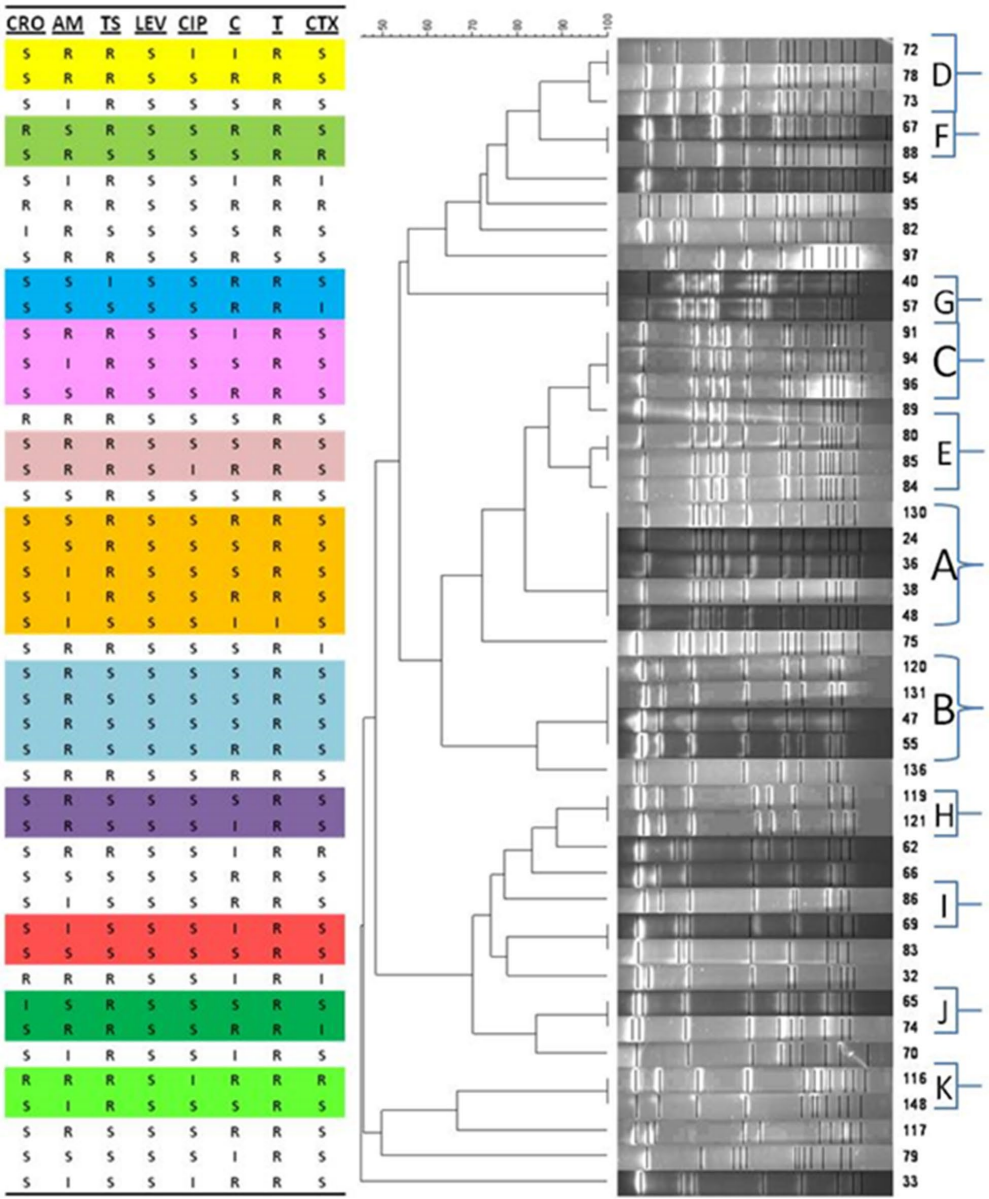
Genomic patterns of nasopharyngeal H. *influenza* isolates by PFGE. Randomly analysed strains PFGE pattern was assessed by a CHEF-DR III software. As seen in Fig. 1 despite different antibiotic resistance pattern of ampicillin resistant isolates, these strains clustered in a similar clone. This also applies in the case of strains resistant and susceptible to chloramphenicol

For PFGE analysis, 45 susceptible and resistant strains were randomly selected among nasopharynx strains. Considering a 90 percent similarity, 28 PFGE patterns were achieved among which 11 patterns included at least 2 strains. The strains clustered into 25 different clones. Clone A had 5 identical strains (strains No. 130, 24, 36, 38, and 48). Clone B had 4 identical strains (strains No. 120, 131, 47, and 55). Clone C, D and E contains 3 strains. And finally, clones F, G, H, I, J and K included two strains. Each of the remaining strains formed a separate clone (Fig. 2 and Additional files 3, 4).

## Discussion

*Haemophilus influenzae* is one of the most important reason of pneumonia and meningitidis in children under 6 years old and the sixth leading cause of death in developing countries, highlighting on the importance of this bacterium in medicine [22, 23]. In treating the patients the antibiotic resistance pattern of this bacterium has been widely studied in recent years. In 2010, Bae et al. by investigation respiratory tract *H. influenzae* isolates in Korea reported the percentage of resistance as 58.5%, 23.3%, 18.7%, 17%, 10.4% and 81% relative to ampicillin, ceforoxim, clarithromycin, cefaclor, amoxicillin, and chloramphenicol respectively [24]. In 2015, Boroumand et al. by investigation of *H. influenza* isolates from Milad hospital in Tehran reported that resistance to clindamycin, chloramphenicol and tetracycline was observed in all (100%) isolates. Percentage resistance to amoxicillin, ceftriaxone, ciprofloxacin and azithromycin were 95%, 50%, 45% and 5% respectively. Also, all isolates (100%) were sensitive to trimethoprim/sulfamethoxazole [25]. However, levofloxacin and cefotaxime had the strongest effect in this study. Within 20 years, Ladhani et al. identified 6805 strains of *H. influenzae* from CSF. More than half of them (55%) were type B. Resistance to ampicillin, trimethoprim, tetracycline, and chloramphenicol were 16%, 8%, 2%, and 1% respectively. All strains were susceptible to cefotaxime and only 0.06% was resistant to rifampin. Based on the results of them research, rifampicin and cefotaxime have the most effect on *H. influenzae* isolates. This shows the changes on antibiotic susceptibility among the strains of *H. influenzae* [26]. In Iran the results of susceptibility of *H. influenza* showed 85% of the isolates were susceptible to chloramphenicol and 71% to ampicillin and cefotaxime [27]. Considering Li et al. findings in 2017 indicated that all respiratory isolates were high resistant to ampicillin, cefuroxime, clarithromycin, and sulfamethoxazole-trimethoprim. The percentage of resistance to cefuroxime, ampicillin/sulbactam, cefotaxime, clarithromycin, and sulfamethoxazole-trimethoprim were 72.1%, 95.9%, 96.4%, 81.8%, and 36.4%, respectively and [28]. In our study, 90 percent of strains were susceptible to tetracycline. Compare to other countries, this level has been increased. The resistance to chloramphenicol and ampicillin were 42% and 43%, respectively which is similar to other countries. In terms of co-trimoxazole, 58% of strains were resistant. This finding is similar to Malaysia [29].

On the other hand, due to the pathogenic importance, *H. influenzae* has widely considered in epidemiologic studies on genetic similarities [19]. Given the dendrogram of the studies strains (Fig. 2), it is observed that a number of strains categorized in one clone (e.g. clone A and B). As the studied population was children under 6 years old from different centers, it is concluded that these strains circulated among children. The strains of clone A, isolated from five different children, are identical in terms of resistance to antibiotics except chloramphenicol. As the same the strains of clone B have identical resistance, except for chloramphenicol which is different in one strain. Regardless of intermediate resistance, the strains of clones D, E, F, G, and H have the same resistance pattern. The strains of clones I and J are partially different in their resistance. Detailed data was specified in Fig. 2 and Additional files 3, 4.

The resistance pattern of one strain in clone B is identical to other strains of that clone except chloramphenicol. In terms of clone C, the strain (strain No. 89) that closely related to other strains, is different from them in resistance pattern. This may be because of the difference in the genetic algorithm. In the strains forming clone A and B, susceptibility to ceftriaxone, levofloxacin, ciprofloxacin and cefotaxime was observed. This showed that these antibiotics have a good therapeutic effect on these strains.

As the studied strains showed a less percentage of resistance to these antibiotics, these drugs can be a good choice for Haemophilus-related infections. Considering chloramphenicol and ampicillin resistant samples, the strains resistant to ampicillin are more identical in terms of genetic similarities and showed more clones comparing with strains resistant to chloramphenicol. This may mean that strains resistant to ampicillin are more resistant to common treatment than strains resistant to chloramphenicol (Fig. 2).

## Limitations

In fact, the patterns of antibiotic resistance did not show a significant correlation with the clonality of the strains. At the current study typing of encapsulated *H. influenza* was not accomplished. It seems that more research is needed to determination of *H. influenza* type strains and find the relationship between clonality and resistance of bacteria to antibiotics.

## Supplementary information

**Additional file 1.** Semi PCR optimization to 350 bp product representative to *omp6* encoded gene. M, DNA ladder 100 bp; 1, *H. influenzae* ATCC 9007 as positive control; 2–10 suspected colonies; 11, *P. aeruginosa* ATCC 27853 as negative control.

**Additional file 2.** Minimum inhibitory concentration of Ampicillin and chloramphenicol resistant and intermediate discriminated strains after Kerby-Baouer procedure.

**Additional file 3.** Antimicrobial susceptibility of 45 randomized nasopharyngeal isolated H*. influenzae* strains and its relatedness patterns in Thran, Iran. Antibiotic susceptibility and dominant PFGE clonal identity of isolated strains. When 45 randomly selected susceptible and resistant isolates were analysed by PFGE, the 28 profiles were observed. Considering 90 percent similarity index, the computer-generated dendrogram showed that out of 28 patterns, 11 PFGE patterns consisted two or more strains. Among Hib isolates, five clones (F–J) were screened and each clone belonged to the two strains. The remaining strains were each identified as a separate clone.

**Additional file 4.** Genetic relatedness of 23 ampicillin-resistant and 18 chloramphenicol resistant *H. influenzae* isolates by cluster analysis of the PFGE patterns. (A) PFGE patterns of SmaI-digested chromosomal DNAs of the Ampicillin resistant (A) and chloramphenicol resistant (B) *H. influenzae* isolates. Strain code numbers and serotype designations are indicated above lanes. M, Lambda ladder PFGE marker (New England BioLabs). (b) Dendrogram based on PFGE SmaI restriction pattern analysis. On the right, the strain code number and the serotype are reported. Similarity analysis was performed with Dice’s coefficient and clustering by the UPGMA method. Isolates with a coefficient of similarity value of ≥ 0.9 were considered to belong to the same clonal group.

## Data Availability

All the results of this study have been classified and maintained by the dissertation in the Pasteur Institute of Iran. We have indeed provided all raw data on which our study is based.
