# Supplementary figures and images for: Clonal diversity of Haemophilus influenzae carriage isolated from under the age of 6 years children
Source: BMC Res Notes. 2019 Sep 11;12:565. doi: 10.1186/s13104-019-4603-7 (PMC6737650; doi:10.1186/s13104-019-4603-7)

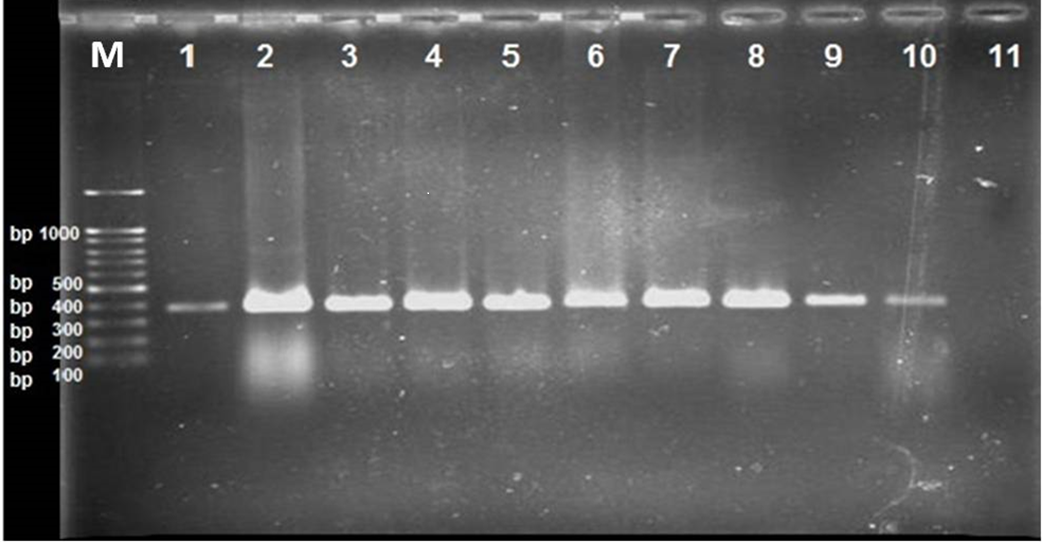

Supplement: Supplementary file 1 — Additional file 1. Semi PCR optimization to 350 bp product representative to omp6 encoded gene. M, DNA ladder 100 bp; 1, H. influenzae ATCC 9007 as positive control; 2–10 suspected colonies; 11, P. aeruginosa ATCC 27853 as negative control. [file 13104_2019_4603_MOESM1_ESM.tif]

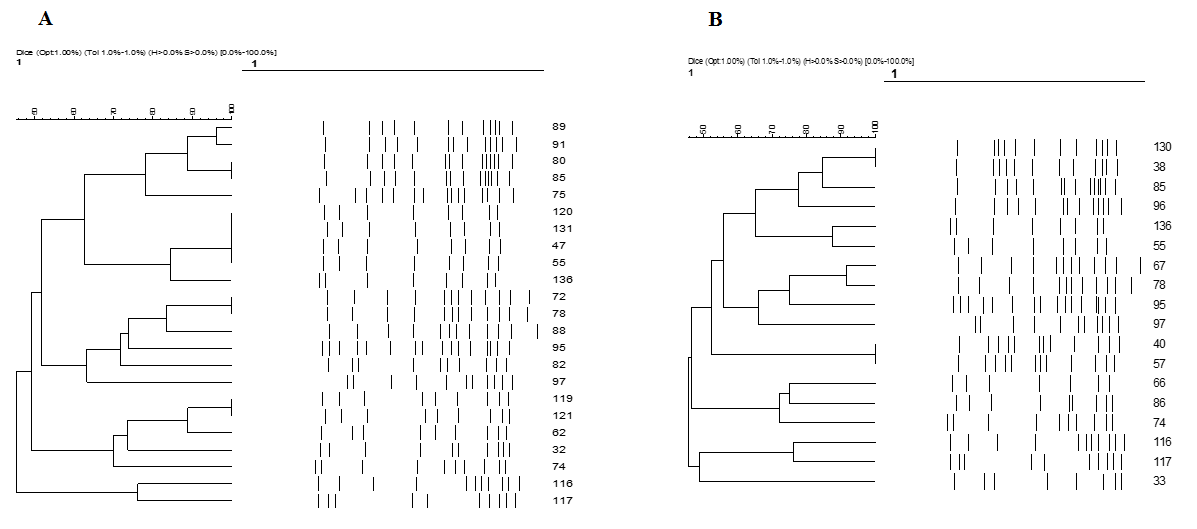

Supplement: Supplementary file 4 — Additional file 4. Genetic relatedness of 23 ampicillin-resistant and 18 chloramphenicol resistant H. influenzae isolates by cluster analysis of the PFGE patterns. (A) PFGE patterns of SmaI-digested chromosomal DNAs of the Ampicillin resistant (A) and chloramphenicol resistant (B) H. influenzae isolates. Strain code numbers and serotype designations are indicated above lanes. M, Lambda ladder PFGE marker (New England BioLabs). (b) Dendrogram based on PFGE SmaI restriction pattern analysis. On the right, the strain code number and the serotype are reported. Similarity analysis was performed with Dice’s coefficient and clustering by the UPGMA method. Isolates with a coefficient of similarity value of ≥ 0.9 were considered to belong to the same clonal group. [file 13104_2019_4603_MOESM4_ESM.tif]
