# Supplementary material for: Clonal diversity of Haemophilus influenzae carriage isolated from under the age of 6 years children
Source: BMC Res Notes. 2019 Sep 11;12:565. doi: 10.1186/s13104-019-4603-7 (PMC6737650; doi:10.1186/s13104-019-4603-7)
Supplement: Supplementary file 2 — Additional file 2. Minimum inhibitory concentration of Ampicillin and chloramphenicol resistant and intermediate discriminated strains after Kerby-Baouer procedure. [file 13104_2019_4603_MOESM2_ESM.docx]

Minimum inhibitory concentration of Ampicillin and chloramphenicol resistant and intermediate discriminated strains after kerby-baouer procedure.

| **Antibiotic** | **Ampicillin** | | | **Chloramphenicol** | | |
| --- | --- | --- | --- | --- | --- | --- |
|  | MIC≤1  S | MIC₌2  I | MIC≥4  R | MIC≤2  S | MIC₌4  I | MIC≥8  R |
| Number of Strains (%) | 38(66.7%) | 3(5.3%) | 16(28%) | 47(96%) | 1(2%) | 1(2%) |

MIC, Minimum inhibitory concentration; R, resistant; I, intermediate; S, susceptible.
