# Supplementary material for: Clonal diversity of Haemophilus influenzae carriage isolated from under the age of 6 years children
Source: BMC Res Notes. 2019 Sep 11;12:565. doi: 10.1186/s13104-019-4603-7 (PMC6737650; doi:10.1186/s13104-019-4603-7)
Supplement: Supplementary file 3 — Additional file 3. Antimicrobial susceptibility of 45 randomized nasopharyngeal isolated H. influenzae strains and its relatedness patterns in Thran, Iran. Antibiotic susceptibility and dominant PFGE clonal identity of isolated strains. When 45 randomly selected susceptible and resistant isolates were analysed by PFGE, the 28 profiles were observed. Considering 90 percent similarity index, the computer-generated dendrogram showed that out of 28 patterns, 11 PFGE patterns consisted two or more strains. Among Hib isolates, five clones (F–J) were screened and each clone belonged to the two strains. The remaining strains were each identified as a separate clone. [file 13104_2019_4603_MOESM3_ESM.docx]

**Additional file 3:**

| **Antibiotics resistant patterns** | | | | | | | | **PFGE**  **Clonal identity** | **Nasopharyngeal isolated strains**  **(number identity)** |
| --- | --- | --- | --- | --- | --- | --- | --- | --- | --- |
| **CRO** | **AM** | **TS** | **LEV** | **CIP** | **C** | **T** | **CTX** |  |  |
| **S** | **S** | **R** | **S** | **S** | **R** | **R** | **S** | **A** | **24** |
| **S** | **S** | **R** | **S** | **S** | **S** | **R** | **S** | **A** | **36** |
| **S** | **I** | **R** | **S** | **S** | **S** | **R** | **S** | **A** | **38** |
| **S** | **I** | **R** | **S** | **S** | **R** | **R** | **S** | **A** | **48** |
| **S** | **I** | **S** | **S** | **S** | **I** | **I** | **S** | **A** | **130** |
| **S** | **R** | **S** | **S** | **S** | **S** | **R** | **S** | **B** | **47** |
| **S** | **R** | **S** | **S** | **S** | **S** | **R** | **S** | **B** | **55** |
| **S** | **R** | **S** | **S** | **S** | **S** | **R** | **S** | **B** | **120** |
| **S** | **R** | **S** | **S** | **S** | **R** | **R** | **S** | **B** | **131** |
| **S** | **R** | **R** | **S** | **S** | **I** | **R** | **S** | **C** | **89** |
| **S** | **I** | **R** | **S** | **S** | **S** | **R** | **S** | **C** | **91** |
| **S** | **S** | **R** | **S** | **S** | **R** | **R** | **S** | **C** | **94** |
| **R** | **R** | **R** | **S** | **S** | **S** | **R** | **S** | **C** | **96** |
| **S** | **R** | **R** | **S** | **I** | **I** | **R** | **S** | **D** | **72** |
| **S** | **R** | **R** | **S** | **S** | **R** | **R** | **S** | **D** | **73** |
| **S** | **I** | **R** | **S** | **S** | **S** | **R** | **S** | **D** | **78** |
| **S** | **R** | **R** | **S** | **S** | **S** | **R** | **S** | **E** | **80** |
| **S** | **R** | **R** | **S** | **I** | **R** | **R** | **S** | **E** | **84** |
| **S** | **S** | **R** | **S** | **S** | **S** | **R** | **S** | **E** | **85** |

CTX; cefotaxime, T; tetracycline, C; chloramphenicol, CIP; ciprofloxacin, LEV; levofloxacin, TS; trimethoprim-sulfamethoxazole, AM; Ampicillin, CRO; ceftriaxone, R; resistant, I; intermediate, S; Susceptible
